# From Chatbots to Co‐Scientists: The Impact of Knowledge‐Generating AI (AI 4.0) on Healthcare and Research

**DOI:** 10.1002/advs.76484

**Published:** 2026-07-07

**Authors:** Weida Liu, Gary Peltz

**Affiliations:** ^1^ Department of Anesthesiology Pain and Perioperative Medicine Stanford University School of Medicine Stanford California USA

## Abstract

While artificial intelligence (AI) has developed as a computational concept for 70 years, its transformative impact on biomedical research and healthcare has explosively accelerated over just the last five years. We are now standing at the precipice of a profound paradigm shift: The emergence of fourth‐generation, knowledge‐generating AI (AI 4.0). Unlike its predecessors, which primarily synthesized existing information, AI 4.0 possesses advanced reasoning and multi‐agentic capabilities that elevate it from a passive tool to an autonomous “co‐scientist.” By independently formulating novel, testable hypotheses, AI 4.0 promises to catalyze biomedical discoveries, dramatically accelerate the development of new treatments, and redefine patient care. In this perspective, we trace the evolutionary arc of AI through its first three generations to contextualize this historic leap. We then explore the transformative potential of AI 4.0 across drug repurposing, genomic medicine, and scientific peer review. Finally, we highlight a critical emerging bottleneck: The urgent need to overhaul our traditional laboratory infrastructure to keep pace with the sheer volume of AI‐generated scientific insight.

## Graphical abstract

This perspective contrasts the historical, linear progression of early AI with the dynamic, iterative nature of AI 4.0; and it describes the real‐world medical applications and the necessary evolution of laboratory infrastructure brought about by AI 4.0.

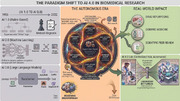

To fully appreciate the revolutionary nature of AI 4.0, it is essential to view its development through the lens of its preceding epochs (Figure 1) [1, 2]. As Howell et al. [3] described, the first “rules‐based” era (AI 1.0) relied on expert‐encoded “if‐then” logic and decision trees. While rigidly constrained by human programming, these early algorithms successfully demonstrated that machines could perform complex cognitive tasks, such as playing grandmaster‐level chess or aiding in early medical diagnoses [4]. Crucially, this period birthed the foundational concepts of modern computing: The idea that a machine could learn from experience as a perceptron [5] and that complex tasks could be executed by large recursive neural networks (RNNs) [6].

**FIGURE 1:**
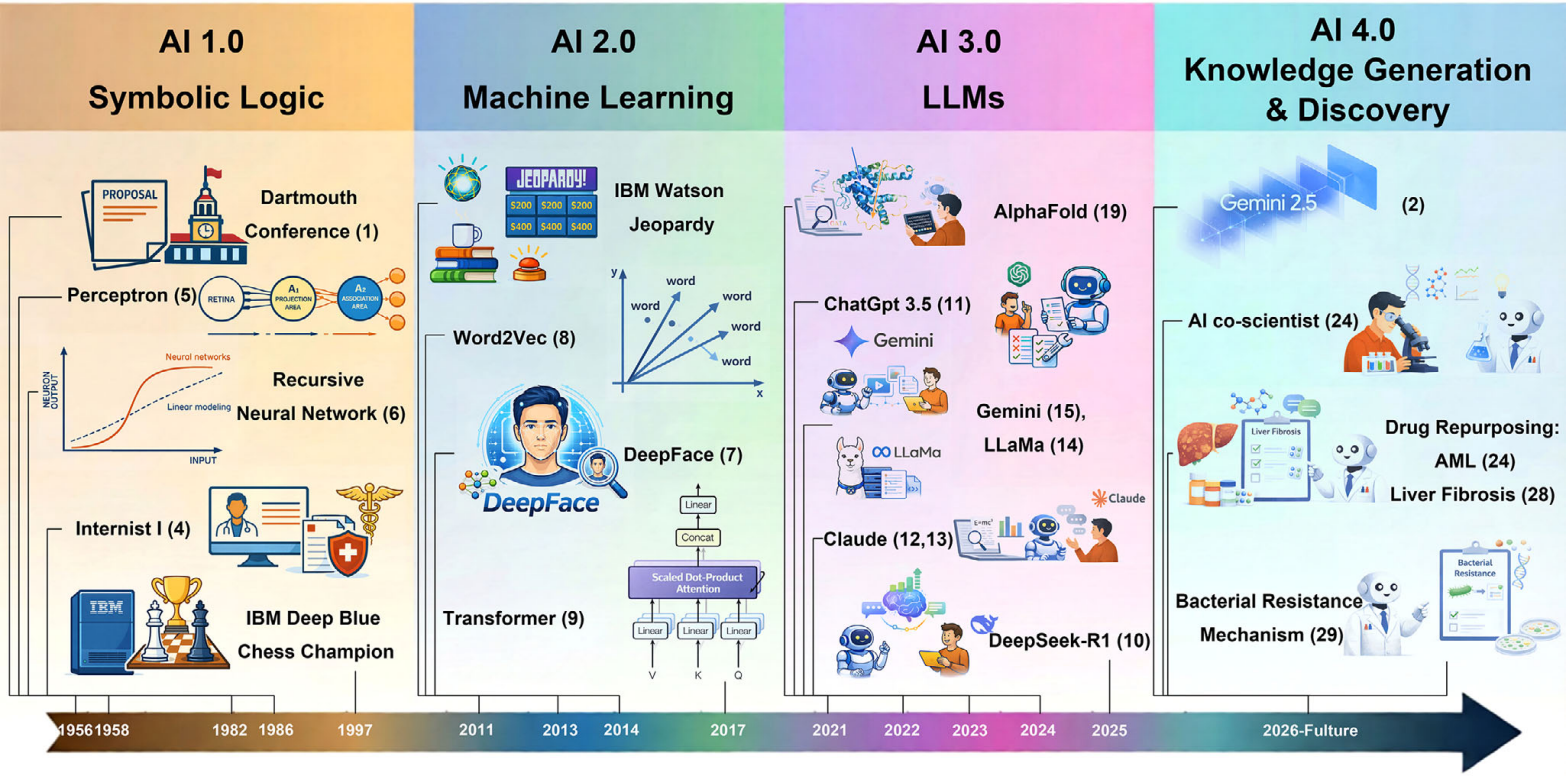
A diagrammatic timeline showing the four AI eras. The key developments occurring within each era are indicated, and the associated references are provided within parentheses. The first AI era (AI 1.0) was initiated by a conference paper, and the foundational papers for machine learning appeared during this era. AI 1.0 programs consisted of algorithms that used expert‐encoded "if‐then" or "decision tree" logic to perform tasks. During AI 2.0, machine learning methods that could perform more complex tasks were developed. AI 2.0, programs were fundamental for developing LLMs, which included those used for facial recognition, converting words to vectors, and transformer architecture. During AI 3.0, multiple LLMs and protein structure prediction methods were released. We are now beginning a new AI era (AI 4.0) that is characterized by LLMs that use multiple asynchronous agents and other tools, which have advanced reasoning capabilities. One AI 4.0 (AI co‐scientist) could generate novel hypotheses that can lead to new discoveries. Three of its novel hypotheses (drug repurposing for AML and liver fibrosis, and a bacterial antibiotic resistance mechanism) were experimentally verified.

Unlike standard networks that process data in a straight line, RNNs process data that is structured as trees or hierarchies by repeatedly applying the same set of mathematical weights over varying parts of a structured input to build a complete representation. The subsequent “machine learning” era (AI 2.0) shifted the paradigm from explicit rule‐following to dynamic pattern recognition. By training multilayered neural networks on vast “ground truth” datasets, AI 2.0 unlocked breakthroughs in medical image classification for retinal and cancer screening [7]. Furthermore, it established the mathematical methods for translating large volumes of words into vectors [8] and introduced the revolutionary transformer architecture [9], providing the critical foundation for the modern AI explosion. Instead of processing data sequentially, transformers use a mathematical mechanism called “self‐attention” to evaluate how every single piece of input data relates to every other piece, regardless of their distance from one another. This allows the model to capture deep context and long‐range dependencies, which enables it to synthesize vast amounts of scientific literature or mod complex genomic sequences.

The convergence of transformer architectures and exponentially increased computational power ushered in the Large Language Model (LLM) era (AI 3.0) in 2022. AI transitioned into highly versatile [10, 11, 12, 13, 14, 15]. In healthcare, these models rapidly demonstrated near‐human proficiency—passing medical licensing exams [16], summarizing patient charts to assist with differential diagnoses [17], and answering intricate health inquiries [18]. Perhaps most spectacularly, AI 3.0 systems like AlphaFold [19] decoded the three‐dimensional structures of proteins, radically accelerating drug and vaccine development. Today, AI 3.0 is seamlessly integrating into clinical workflows, particularly in image‐heavy fields like pathology [20, 21] and radiology [22, 23]. Yet, despite its profound utility, AI 3.0 remains fundamentally reactive. Operating via a single, rapid computational pass, it relies on static training data and lacks the capacity to independently reason, self‐correct, or generate entirely new scientific frameworks. Consequently, human physicians and researchers must remain squarely “in the loop” to synthesize outputs and drive actual scientific discovery.

That critical limitation in reasoning was shattered in 2025 with the advent of AI 4.0—a new class of knowledge‐generating AI equipped with enhanced reasoning and autonomous, multi‐agentic capabilities [2]. Unlike its predecessors, AI 4.0 breaks free from the single‐pass constraint by scaling up and optimizing the “test‐time compute” paradigm [24, 25, 26], granting the system the time and computational resources to “think,” reason, and build upon its training data before providing a response. Exemplified by the multi‐agent “AI co‐scientist” (built on Gemini 2.0) [27], these systems execute a dynamic “generate, rank, and evolve” workflow to uncover new knowledge and formulate actionable research hypotheses (Figure 2). This system is composed of multiple, interacting AI “agents.” Each agent is given a specific role, instruction set, or tool (e.g., one agent analyzes data sets, another synthesizes hypotheses, and a third acts as a critical reviewer). These agents collaborate, iterate, and debate to execute highly complex, multi‐step workflows. This framework is the operational foundation for developing autonomous “AI co‐scientists” that can independently navigate research environments. By deploying specialized, asynchronously functioning agents that gather new data via external tools like web search, verify facts, and debate findings, AI 4.0 iteratively refines its logic and catches its own mistakes, drastically reducing hallucinations. Its staggering potential to catalyze discoveries has already been validated in the laboratory: AI co‐scientist successfully formulated novel, experimentally verified hypotheses for drug repurposing in liver fibrosis [28] and acute myelocytic leukemia (AML) [27], and uncovered a groundbreaking mechanistic explanation for the rapid spread of antimicrobial resistance among bacteria [29].

**FIGURE 2:**
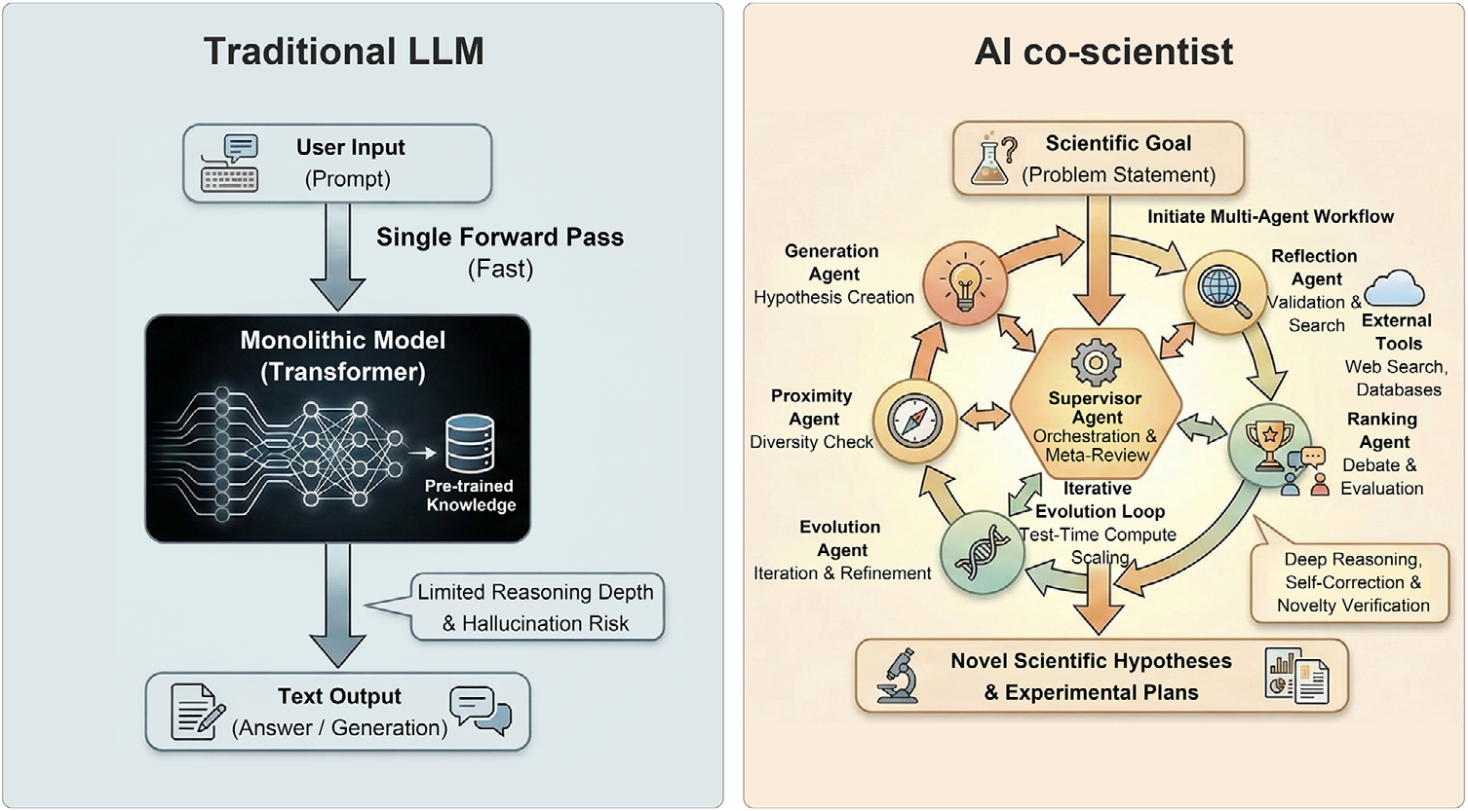
Architectural and algorithmic advances utilized by an AI 4.0 (AI co‐scientist). A traditional (AI 3.0) LLM has a linear workflow (input leads to output) that provides a rapid, single‐pass analysis based upon a transformer and its training data (i.e., its knowledge is static); and its output has limited reasoning depth and no self‐correction, which creates a risk for hallucination. AI co‐scientist is a compound, multi‐agent system that can evaluate a complex scientific problem and produce novel hypotheses, and can generate plans for their experimental validation. AI co‐scientist uses an iterative loop where its various agents function in an asynchronous manner and utilize scaled test‐time compute to generate hypotheses, which are ranked, evaluated (for novelty and other features), and then refined. The hypotheses are then evaluated and grounded using external tools (web search and databases). Because of its evolving and self‐correcting features, AI co‐scientists' generated novel hypotheses have scientific rigor, along with the plans for their experimental validation.

Although we cannot predict all the impact that AI 4.0 will have on healthcare, there are three areas where it will introduce revolutionary changes in the near term. LLMs can be used to accelerate the discovery and development of new drugs [30], but we predict that a major impact of AI 4.0 will be to accelerate drug repurposing for the treatment of other diseases. Drug repurposing can produce new treatments at a much faster rate than the 12 years that are now required for the discovery, characterization, and clinical testing of new drugs [31]. However, repurposing a drug for even one disease requires a comprehensive understanding of the pathobiology of a disease, extensive knowledge about possible therapeutic targets for it, and an understanding of the effects that each of the ∼20 000 FDA‐approved drugs marketed for the treatment of other diseases [32] can have on disease‐causing pathways. There have been prior attempts at computational drug repurposing, but they used knowledge graphs, graph convolutional networks [33, 34], or graph‐based computational approaches [35]. However, the prior approaches lack scalability, have a very limited ability to make complex connections between drugs and disease biology, fail to explain the drug‐disease connections, and cannot design experimental testing methods. None of the drugs identified by these methods have yet been experimentally tested for their effect in a disease model. In contrast, AI co‐scientist can make complex connections between drug effects and disease biology, provide the rationale underlying those connections, and develop plans for testing the effect of a recommended drug on disease biology. It is noteworthy that two of the three repurposed drugs recommended by AI co‐scientist for liver fibrosis exhibited antifibrotic activity when tested, while neither of the two drugs selected by the principal investigator exhibited antifibrotic activity. Moreover, in addition to their antifibrotic activity, the AI‐repurposed drugs also promoted liver cell regeneration, and one was an FDA‐approved drug for the treatment of another disease [28]. Hence, AI‐based drug repurposing could initiate a new class of antifibrotic therapies that also promote liver regeneration. It is likely that AI co‐scientist (or other AI 4.0 systems) will be a part of larger efforts to repurpose drugs for the estimated 17 080 known human diseases [36], the vast majority of which (90%–95%) have no treatments.

While AI 4.0 can synthesize vast amounts of data and generate hypotheses at an unprecedented rate, this creates a new scientific bottleneck that must be overcome. While AI 4.0 can generate 100 plausible drug repurposing hypotheses very quickly, we currently have a very limited capacity for experimental testing of these hypotheses. Experimental validation is the centerpiece of biomedical science; hypotheses without experimental validation are of little value. To truly take advantage of AI 4.0's data synthesis and hypothesis‐generating capabilities for drug repurposing, the scientific community will need parallel advancements in automated (“robotic”) wet lab capabilities that will enable the large volume of AI 4.0‐generated ideas to be experimentally tested with human‐based systems. The ability to grow human liver organoids in microwells made it possible to quickly test the drugs that AI co‐scientist suggested for repurposing for liver fibrosis [28], and this provides an example of a technology that could be adapted for the many diseases that can be modeled with human organoids. Similarly, human organ‐on‐a‐chip methods have rapidly advanced to enable experimental systems for drug efficacy testing for many different types of diseases. Customized microelectrode arrays and human stem cells have produced human‐on‐a‐chip systems used for drug efficacy testing for many diseases, which include Alzheimer's disease [37, 38, 39, 40], amyotrophic lateral sclerosis [41, 42], and Charcot–Marie–Tooth disease [39].

Just as AI has accelerated the pace of genetic discovery for mouse models [43], the second near‐term area of AI 4.0 impact is on genetic discovery and genetic disease diagnosis. We recently demonstrated that a LLM could analyze entire genomic sequences obtained from inbred mouse strains and identify a genetic factor contributing to the spontaneous hearing loss occurring in one inbred strain, which was experimentally validated by characterization of knockin mice [44]. Based upon the mouse findings, we developed a pipeline that enabled an advanced LLM (Gemini 2.5 Pro) to analyze large lists of genes, which contained the genetic variants present in the genomic sequences of 20 human subjects with hearing loss, and it identified human causative genetic factors for their hearing loss. Otolaryngologists agreed with 18 of the 20 variants identified by the AI. This AI also was able to identify causative genetic factors for six subjects with rare genetic diseases, which required 14–34 different terms to describe their multi‐faceted symptom complexes [44]. Hence, an AI pipeline has already been shown facilitate genetic diagnosis and discovery in mice and humans.

In the above examples, the AI was provided with the lists of genetic variants and diagnoses for each patient. Soon, the AI pipeline will be able to semi‐autonomously identify causative genetic factors based upon its own analysis of a patient's medical record and of raw genomic sequence data. If so, AI‐based methods could efficiently determine the genetic basis for the estimated ∼350 million people globally [45] with suspected genetic diseases [46] or rare undiagnosed syndromes [47]. More broadly, since an increasing number of individuals will have their genome sequenced, AI could improve healthcare for billions of individuals by providing them with access to precision genomic health. This could allow people to access definitive therapies in a timely manner and enable individuals to better understand the impact of their genetic determinants on their health. This could be transformative for public health: Medical practice could shift from its current focus on disease treatment toward disease prevention; customized plans for disease prevention could be developed based upon genetic factors. Of importance, this would enable individuals living in regions that do not have geneticists or genetic counselors to benefit from the information contained in their genomic sequence. However, FDA regulation is a major bottleneck for making full use of AI 4.0 for genetic diagnosis. The FDA's currently regulates AI 3.0 (i.e., algorithms that perform a limited set of tasks) under the Software as a Medical Device framework [48, 49]. It remains to be determined how (and even if) the FDA can regulate autonomously functioning AI 4.0 agents that can dynamically reason and produce output that can evolve as the available information changes.

Peer review is an area that AI has already impacted. The turnaround time required for grant applications and publication review has been greatly elongated since the COVID‐19 era, which has had a chilling effect on the pace of biomedical discovery. Moreover, due to the highly competitive nature of obtaining the limited amount of available funding, “tribalism” has set in among reviewers because the journals and granting agencies select reviewers who are working in the areas covered by the submitted papers or grant applications. However, it is these reviewers who are most likely to favor submissions by investigators that are aligned with them and to be much less favorable to investigators who are not. AI can overcome the time and bias problems that have plagued peer review and could improve review quality. Knowledge‐generating AIs can summarize the findings and assess novelty, methodology, and potential impact in a fraction of the time required by a human reviewer. Although the use of AI for review purposes is currently discouraged by journals and granting agencies, a recent survey indicates that 50% (of 1600 scientists surveyed) are using AI for preparing reviews [50, 51]. Hence, AI use for peer review has already reached a tipping point. Using AI could increase the quality, timeliness, and fairness of peer review, which will make a major contribution to biomedical research. However, there are issues with using AI for peer review that must be overcome. Since LLMs are trained on the existing literature, an AI may reject novel, paradigm‐shifting ideas, which would simply substitute algorithmic conservatism for tribalism. There are also confidentiality concerns when unpublished grants or papers are placed on commercial LLMs for review. The confidentiality concern could be overcome by creating secure, locally hosted LLMs designed for paper or grant review that are hosted by the granting agencies or journal publisher.

*Future directions*: While AI 4.0 will undoubtedly revolutionize healthcare and biomedical research, its full impact likely extends far beyond what we can currently envision. To fully harness these emerging capabilities; however, fundamental changes to our organizational structures and training programs are required. First, medical and scientific training programs must adapt to an AI‐integrated world. Medical education must shift away from rote memorization, while scientists must learn to effectively collaborate with autonomous systems. Mastering iterative inquiry, strategic goal specification, and workflow orchestration will be essential for guiding AI 4.0 to produce novel hypotheses. Second, clinicians and scientists must develop the critical skills necessary to evaluate complex, multi‐agent reasoning—whether for genetic discovery or disease diagnosis—and to order laboratory tests or design the precise experiments required to validate AI‐generated ideas. Third, and most critically, the traditional architecture of the experimental laboratory must evolve. The prevailing model, which is typically centered on a single investigator with limited automation, lacks the throughput required to keep pace with rapid AI hypothesis generation. Moving forward, research institutions must transition toward highly automated, centralized laboratory hubs designed to service multiple investigators simultaneously (Figure 3). Of note, the concept that a computer‐driven lab could function as an “automated scientist,” which could generate and experimentally test functional genomic hypotheses, was demonstrated over 15 years ago [52]. Although restructuring these entrenched paradigms will be challenging, creating physical environments, and training programs that are optimized for AI 4.0 will dramatically accelerate the pace of scientific discovery and improve healthcare in the 21st century.

**FIGURE 3:**
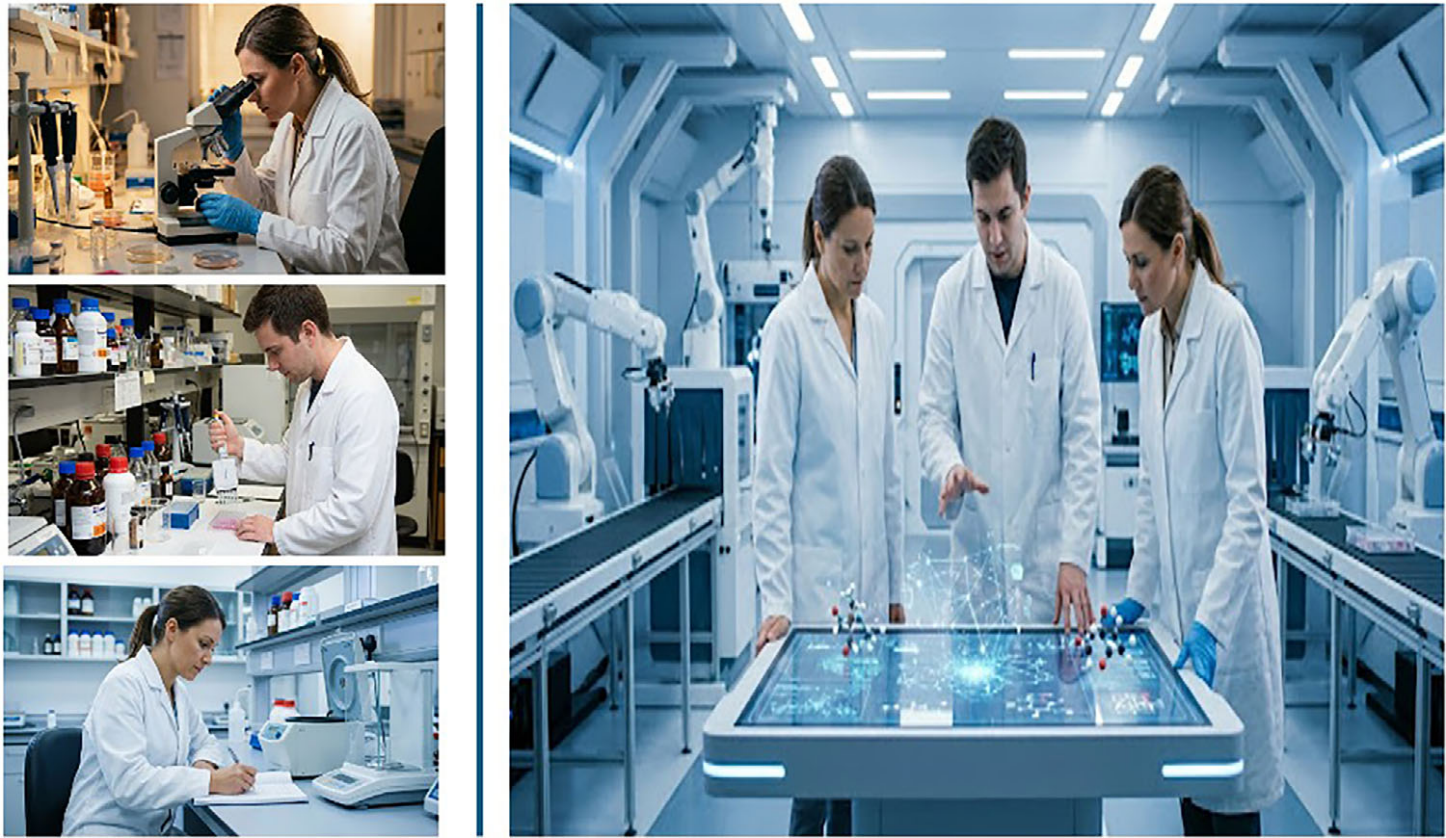
Comparison of current research laboratory configurations (left) with a laboratory designed to make optimal use of AI 4.0 knowledge‐generating capabilities (right). Currently, research often occurs in siloed configurations with individual investigators performing manual benchwork (Left panels). An AI 4.0 lab, however, will be designed as a centralized hub to serve multiple collaborating investigators (right). It will feature extensive automation with the throughput required to experimentally test the vast volume of hypotheses generated by AI 4.0 models. (Images generated by Nano Banana).

## Author Contributions

G.P. formulated the concept, and W.L. and G.P. wrote the paper.

## Conflicts of Interest

The authors declare no conflicts of interest.

## Data Availability

The authors have nothing to report.
